# Perioperative management of a patient with subcutaneous defibrillator undergoing cardiac surgery

**DOI:** 10.1186/s40064-015-1315-x

**Published:** 2015-09-21

**Authors:** Tommaso Infusino, Sergio Valsecchi, Michele Rigano, Daniele Maselli

**Affiliations:** Department of Cardiovascular Surgery, Sant’Anna Hospital, Viale Pio X, 111, 88100 Catanzaro, Italy; Boston Scientific, Milan, Italy

**Keywords:** Subcutaneous defibrillator, Inappropriate shock, Temporary pacing, Sternotomy, Cardiac surgery

## Abstract

We describe a case of inappropriate shocks due to temporary epicardial pacing after cardiothoracic surgery in a patient with a subcutaneous ICD.

A 55-year-old man underwent implantation of a totally subcutaneous defibrillator (S-ICD), for primary prevention of sudden cardiac death (Fig. 1). At implantation, the lead was vertically positioned in subcutaneous tissue of the chest, 2 cm to the left of the sternal midline. Nine months later, the patient underwent combined aortic and mitral valve replacement via median sternotomy. Special attention was paid to avoid direct application of electrocautery directly to the coil. At the end of the procedure, temporary unipolar atrial and ventricular pacing wires were positioned, and connected to an external dual-chamber pacemaker programmed to promote intrinsic rate and atrio-ventricular conduction (DDD pacing mode, 50 beats/min and long atrio-ventricular delay, with atrial and ventricular pacing output set at 10 mA). After confirmation of unaffected sensing of subcutaneous signals, the S-ICD, deactivated during the procedure, was reprogrammed to enable the tachyarrhythmia detection and therapies. On the first day after surgery in the intensive care unit, because of an abrupt drop in arterial pressure, the pacing rate was immediately increased to 80 beats/min and atrial and ventricular pacing output were set to the maximum value of 25 mA with the aim of ensuring capture. Subsequently, the pacing spikes were intermittently detected by the S-ICD and together with R and T waves of paced beat were identified as sensed complexes (Fig. 2), resulting in delivery of an inappropriate shock. The presence of adequate spontaneous heart rate and conduction was confirmed, thus the pacemaker was reprogrammed to inhibit pacing. Subsequently, the postoperative course was uneventful. Before discharge, the S-ICD was interrogated to confirm the adequacy of sensing vector for arrhythmia detection in the final condition. The patient gave his consent to the publication of his personal and medical information. Inappropriate shocks due to temporary epicardial pacing after cardiothoracic surgery have been reported before in patients with traditional transvenous ICD (Gelissen et al. 2010), and we describe the same case with an S-ICD.

**Fig. 1:**
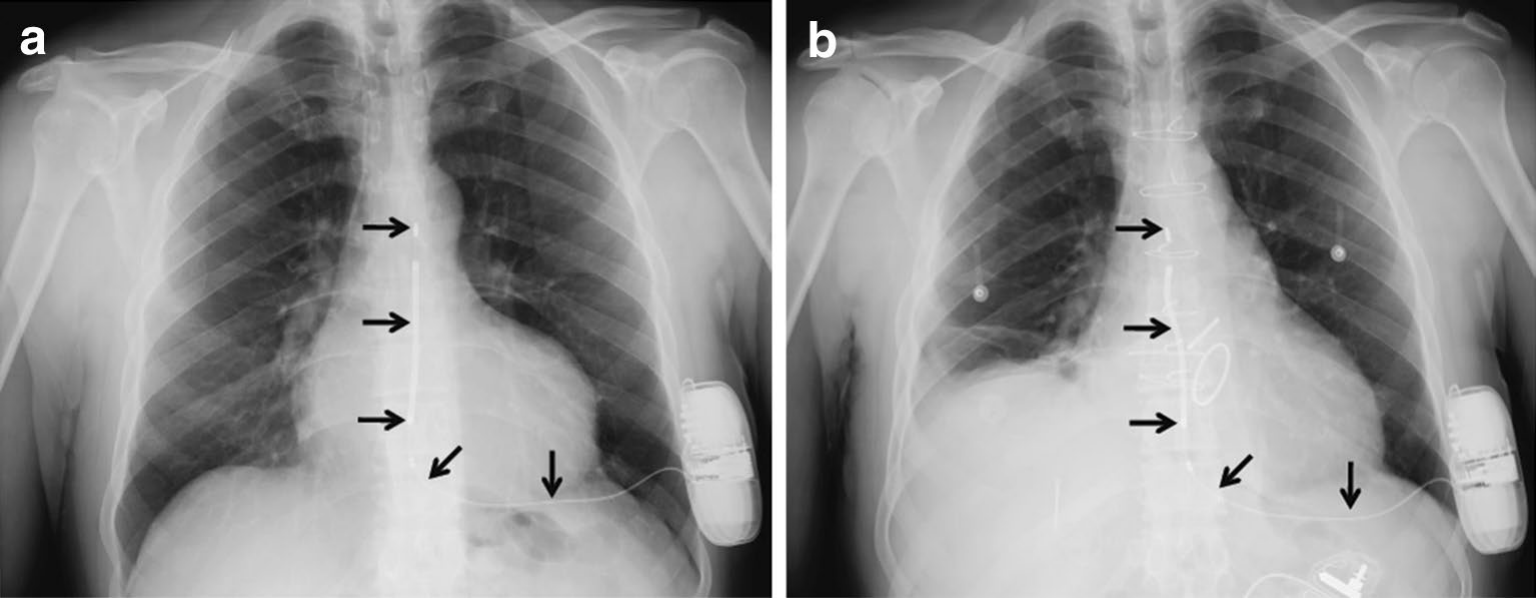
Comparison of chest X-rays before (**a**) and after (**b**) the surgical procedure confirmed an adequate positioning of the lead

**Fig. 2:**
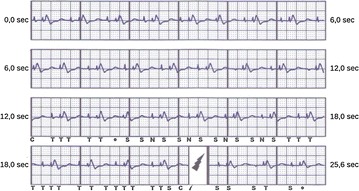
S-ICD electrogram of the episode of inappropriate shock

## Conclusions

The management of an S-ICD during and after surgical procedures is not addressed by current recommendations (Crossley et al. 2011). According to our experience, cardiac surgery through median sternotomy is feasible in the setting of a previously implanted S-ICD system, but care must be taken to avoid cautery application to the lead, and placing the metal sternal wires in contact with the lead. If post-operative pacing is required, it is strongly advised to use bipolar wires and to carefully program the pacemaker (e.g. low pacing output, low pacing rate, single chamber pacing mode, etc.). In addition, appropriate lead location and S-ICD sensing should be reconfirmed postoperatively (considering also possible occurrence of pacing), with consideration of the optimal sensing vector as well.
